# Driving Speeds in Urgent and Non-Urgent Ambulance Missions during Normal and Reduced Winter Speed Limit Periods—A Descriptive Study

**DOI:** 10.3390/nursrep12010006

**Published:** 2022-02-03

**Authors:** Jukka Pappinen, Hilla Nordquist

**Affiliations:** 1 FinnHEMS Research and Development Unit, FI-01530 Vantaa, Finland; 2 Faculty of Health Sciences, University of Eastern Finland, FI-70211 Kuopio, Finland; 3 Department of Health Care and Emergency Care, South-Eastern Finland University of Applied Sciences, Pääskysentie 1, FI-48220 Kotka, Finland; hilla.nordquist@xamk.fi

**Keywords:** traffic safety, driving safety, emergency medical services, driving speed, urgent ambulance missions, non-urgent ambulance missions

## Abstract

Objective: Most traffic research on emergency medical services (EMS) focuses on investigating the time saved with emergency response driving. Evidence regarding driving speed during non-urgent ambulance missions is lacking. In contrast, this descriptive study compared registered driving speeds to the road speed limit in urgent A-missions and non-urgent D-missions. Specifically, the study examined driving speeds during normal speed limits, periods of reduced winter speed limits, and speeding during non-urgent D-missions. Methods: Urgent A-missions and non-urgent D-missions were included. Registered ambulance locations and speed data from Pirkanmaa Hospital District, Finland between 1 January 2018 and 31 December 2018 were used. Ambulance locations were linked to OpenStreetMap digital road network data. The registered driving speed distribution was reported as quartiles by the effective road speed limit. Furthermore, the results during the normal speed limit and reduced winter speed limit periods were reported separately. Driving speeds in non-urgent missions were compared with current Finnish traffic violation legislation. Results: As expected, the urgent A-missions exceeded the speed limits during both the normal speed limit and reduced winter speed limit periods. On the smallest streets with speed limits of 30 km/h, the driving speeds in urgent missions were lower than the speed limit. The driving speeds in non-urgent D-missions were broadly similar throughout the whole year on high-speed roads, and mostly on lower speed limit roads. However, within the 30 km/h speed limits, the mean speed in non-urgent missions appeared to increase during the winter. One-fifth of the registered non-urgent D-missions were speeding. Conclusions: Speeding is common in urgent A-missions and non-urgent D-missions throughout the year. Stricter guidelines for EMS are needed to increase driving safety.

## 1. Introduction

One of the primary traffic safety risk factors is driving speed, and higher speeds increase accident and collision risks [1,2]. A common aim of ambulance missions is to achieve quick access to the target destination [3,4]. However, there is a lack of data concerning the factors affecting the actualized driving speeds in emergency medical services (EMS), and how these speeds compare with speed limits. Emergency response driving permits the use of warning lights, sirens, and exceeding the speed limit. Nevertheless, debates continue regarding whether the clinical benefits of emergency response driving are greater than the increased accident risk for EMS personnel, patients, and other road users [3,5,6,7,8,9,10,11,12,13]. A recent integrative review [3] highlighted that because of the associations with prehospital time, medical care, and safety aspects, the way an ambulance is driven becomes an important part of prehospital care.

In Finland, since 2018, a uniform nationwide method has been used to implement an EMS’s risk classification system. The risk classification is based on 1 km × 1 km areas, which are classified based on the Finnish Environment Institute’s classifications of core urban, other urban, populated rural, and other areas. Moreover, EMS missions are classified into four urgency classes: A to D, where A is the most urgent and D is non-urgent. For the urgent missions (classes A and B), the reach time delays per area are regulated by hospital districts. The reach time is calculated from the time the emergency dispatcher alerts the first EMS unit until the first unit is on site. For example, in the Pirkanmaa Hospital District, 50% of the A-missions must attain a reach time of ≤6 min, and 90% a reach time of ≤10 min in the core urban areas. For the other urban areas, the target reach times are ≤8 min and ≤16 min, and for populated rural areas, ≤15 min and ≤30 min, respectively. Finally, reach times for non-urgent D-missions must be ≤120 min in populated areas for 90% of the missions, and this is uniform nationwide [14]. Notably, ambulances are subject to the same traffic restrictions as other vehicles when undertaking non-urgent missions. Accordingly, it is reasonable to expect that ambulances on D-missions do not need to speed to hit the target reach times.

In Finland, EMS personnel are not professional drivers or even specially trained to drive an ambulance despite driving being a significant part of the role. Driving speed and speeding among general public drivers have been the subject of several studies. A recent meta-analysis concluded that speed is a central risk factor for both accident occurrence and injury severity [15]. One study detailing real-life behavioral data of 3150 volunteers and five million trips in the USA found that mean speeds tend to increase alongside speed limits, but less rapidly in areas with higher speed limits. Furthermore, drivers were found to adapt their speeds based on environmental changes. For instance, drivers reduce their speed limit because of adverse weather conditions, cross-section areas, work zones, and, unsurprisingly, heavy congestion [16]. Moreover, data from a Canadian study with a large real-life speed dataset also noted that on-street parking and the absence of lateral shoulders are associated with restriction compliance [17].

The majority of research examining EMS driving has investigated the time saved with emergency response driving [3,5,6,7,8,9,10,11,12,13]. Lately, however, ambulance driving safety has received increasing attention [18,19]. Recent reviews [3,20] have gathered evidence that time pressure, multitasking, long shifts, and stress have an impact on overall ambulance driving safety. Studies investigating ambulance driving safety [3] typically lack a speeding perspective, especially regarding non-urgent missions. Nonetheless, some evidence exists for urgent missions. A recent Polish study [21] demonstrated that rush hours have no significant impact on ambulance speed during urgent missions, thereby demonstrating that ambulances move at a relatively constant average speed when performing emergency response driving. According to one Finnish study, EMS personnel may show irresponsible and indifferent attitudes when performing emergency response driving [22]. These manifest as excess situational speed, overestimation of driving skills, carefreeness, and carelessness toward other road users [22]. Encouragingly, a recent German study showed that paramedics receiving simulator-based training were positively influenced in terms of the driver’s knowledge, attitudes, and real driving behavior, including reducing speeding [23].

Collectively, the background set by these studies strongly recommends avoiding unnecessary speeding in EMS. However, more research is required to develop evidence-based leadership practices and guidelines for better driving safety in EMS [3,24]. Currently, emergency response driving, and non-urgent mission speed estimates are largely educated guesses or outcomes of commercial routing tool estimates. To support better driving safety and planning in EMS, it is vital that accurate driving speed estimates are based on real ambulance driving speeds. In this study, we aimed to examine (1) registered driving speeds compared to the road speed limit in the most urgent A-missions during normal speed limits and reduced winter speed limit periods. (2) Registered driving speeds compared to the road speed limit in non-urgent D-missions during normal speed limit and reduced winter speed limit periods. Finally, we also examined (3) how many of the non-urgent D-missions exceeded the speed limits set by Finnish traffic violation legislation. In addition to feeding into traffic safety considerations, the findings of this study can be used to assess driving speed for various spatial and logistics analyses.

## 2. Materials and Methods

This is a descriptive study comprising data on registered ambulance locations and speed data from the Pirkanmaa Hospital District between 1 January 2018 and 31 December 2018. Pirkanmaa was chosen as the study area due to its geographical and demographic representativeness, and easy access to the data. The Pirkanmaa Hospital District authorized the study.

Ambulance TETRA-based radio terminals automatically transmit data packets containing the current speed, direction, and location as specified by a pre-determined schedule. These location packets are sent every few hundred meters, yet the exact schedule is not known. In the Pirkanmaa area, speed, and location data are stored in a database for statistical and management purposes. The data for the study were generated by Pirkanmaa Hospital District IT services using a proprietary database query.

The data do not contain information about the mission’s location or nature, the identity of the paramedics, or the unit ID. The time stamps were coarsened to a one-hour accuracy. Only missions with urgency classes A or D, both in response and transport, were included in this study. Urgency class was included with each location data.

Only ambulance units were included in this study. Accordingly, helicopter, and non-transport units (EMS supervisors, emergency physicians, and medical first responders) were excluded. Moreover, the exclusion criteria also included regions with 0 to 5 km/h speeds, as very slow or zero speeds indicate stopping or slow maneuvering (such as parking or turning a vehicle around). Furthermore, inaccuracies in satellite navigation may result in a near zero speed being registered, even for a stationary vehicle.

Each location was connected to OpenStreetMap digital road network data [25] if the location coordinates were within 5 m of the road line. The road type and speed limit were attached to the location. Subsequently, two datasets were created: an urgent dataset for class A-missions (652,759 locations from 50 ambulances) and a non-urgent dataset for class D-missions (141,140 locations from 42 ambulances).

The distribution of registered driving speeds was reported as quartiles by the effective road speed limit. The findings were reported separately for the normal speed limit and the reduced winter speed limit periods (1 January 2018–9 April 2018 and 25 October 2018–31 December 2018, respectively). During the winter, speed limits were reduced on roads from 120km/h to 100km/h. Additionally, driving speeds in the non-urgent dataset were compared with current traffic violation legislation in Finland [26]. MapInfo 17.0 was used for processing geographic data, and SPSS 26.0 was used for statistical analysis.

## 3. Results

Table 1 and Table 2 list the registered driving speeds for urgent ambulance missions and non-urgent ambulance missions, respectively.

During urgent missions, driving both relatively and absolutely in excess of the speed limits took place on roads with 60 or 80 km/h speed limits (Table 1). The speeds were generally lower during the reduced winter speed limit period. Nevertheless, a similar trend was visible during the normal speed limit period. Notably, on streets with a 30 km/h speed limit, both the average and median driving speeds were lower than the speed limit.

During non-urgent D-missions, the driving speeds were broadly similar (Table 2). However, on roads with a 60 and 80 km/h speed limit, the median speed exceeded the speed limit both during the normal speed limit and the reduced winter speed limit periods. In locations with a 30 km/h speed limit, the mean speed appeared to increase during the reduced winter speed limit period compared to the normal speed limit period.

**Figure 1:**
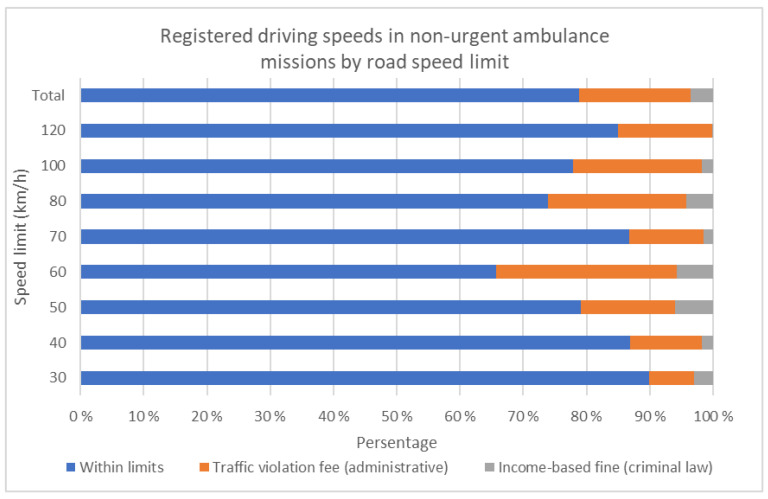
Registered driving speeds in non-urgent ambulance D-missions by road speed limit.

A total of 21.2% of the registered speeds would have resulted in a penalty (Table 3), with 3.6% of them exceeding the criminal fine limit (>20 km/h overspeed). Again, the majority occurred on roads with 60 or 80 km/h speed limits (Figure 1).

## 4. Discussion

We examined registered driving speeds by road speed limit in urgent A-missions and non-urgent D-missions during the normal speed limit and reduced winter speed limit periods. We also examined speeding during non-urgent D-missions. Our primary findings included: (1) Urgent A-missions showed driving speeds that exceeded the speed limits during both normal speed limits and reduced winter speed limit periods. (2) On the smallest streets with a 30 km/h speed limit, the driving speeds in urgent A-missions were lower than the speed limit. (3) In the non-urgent D-missions, the driving speeds were broadly similar throughout the whole year on high-speed limit roads and mostly on lower speed limit roads. (4) In non-urgent D-missions, within the 30 km/h speed limit, the mean speed increased during the winter. Finally, (5) one-fifth of the registered non-urgent D-missions were classed as speeding.

Speeding during urgent EMS missions is common and is expected to a degree, as it is considered a means of reducing response time. The time saved when performing emergency response driving varies from study to study [5,6,7,8,9,10,11,12,13]. Geographical differences are a possible explanation. For instance, Murray et al. [13] noted that studies conducted in more population-dense areas tended to show a greater amount of time saved with emergency response driving compared to studies conducted in rural areas. These findings are in keeping with expectations, as during an emergency response, ambulance driving typically deviates from traffic rules, such as obeying intersection lights or speed limits. Such an impact may be more pronounced in the city than on roads that already dictate high speed limits [16]. Moreover, the finding that the driving speeds on the smallest streets were lower than the speed limits may be associated with the target address search. It can also be linked to drivers demonstrating extra vigilance with the driving environment, as 30 km/h streets tend to go through dense residential areas. It should be noted that this study was conducted in a geographically large hospital district with core urban, other urban, populated rural, and other areas, such as forests with no settlements but many hiking activities. Consequently, the results cannot be easily interpreted based on geographical elements or differences in target reach times of different risk classification areas. Accordingly, this phenomenon should be investigated in greater detail in future studies.

Evidence of driving speed during non-urgent ambulance missions is lacking. Previous studies conducted on the general public have shown that drivers adapt their speed to the environment, such as in cases of adverse weather conditions, drivers were found to reduce their speed [16]. In contrast, a recent study from Poland established that ambulances move at a constant speed regardless of the season [21]. The data collated in this study is limited with respect to examining this association. However, given the current results, this phenomenon was not observed. Thus, it would be interesting to undertake future studies that would investigate whether an association exists between registered ambulance speeds and weather statistics. One such study could examine ambulance speed associations with depth of snow in winter in Finland, which ranges between 10–60 cm and lasts between 100–145 days in the Pirkanmaa area [27]. Currently, this statistic is uncertain. In addition, it may be useful to examine further the increasing speeds during the winter period on 30 km/h roads. This association may require more detailed location studies. However, it is worth noting that this speed limit group had the smallest number of overspeeds. Thus, this finding may not be associated with real-life risk-taking.

One notable finding of this study is that speeding in non-urgent missions is common. There was no clear pattern between the number of overspeeds and the speed limits, as the proportions varied between the speed limit categories. Considering that the EMS personnel in this study were performing work duties and that ambulances are under continuous public scrutiny in traffic, a large number of remarkable overspeeds is concerning. As ambulances on non-urgent missions are subject to the same traffic restrictions as other vehicles, and the non-urgent D-missions should not require speeding in terms of target reach times, this finding hints at irresponsible and indifferent attitudes [22] toward obeying speed limits, even when on duty. Previous evidence [2,3,20,23,24] and findings of this current study demonstrate that there is a need for driver training and stricter driving guidelines in EMS.

A study by Bui et al. established that the risk of a crash increased substantially for every kilometer exceeding the speed limit in non-urgent missions [2]. Concerns need to be raised when considering the speeds recorded in the urgent missions and previous evidence that there might be some undesirable attitudes—at least from a safety perspective—associated with emergency response driving. For instance, these attitudes may be born of a greater sense of power, a likelihood of overtaking whenever possible, risk taking, aggression, overconfidence, frustration, and lack of concentration [20,22,28]. In general, transportation-related injuries constitute a significant proportion of occupational injuries among EMS workers [3,20,24,29,30,31]. Moreover, EMS workers’ indifference toward traffic violation legislation makes a significant contribution to risks [2,32]. Given that ambulance crashes occur during emergency response driving and during normal, non-urgent driving [19,33], there is evidently a clear need to improve risk awareness and driving safety in EMS [3,24].

The results of this study can be employed to estimate an ambulance’s catchment area within a set time or patient transport time within specific regions around hospitals. Nonetheless, to generate an impact on real-life driving safety and guideline implementation, there is still a need for more evidence on EMS mission speed estimations. To gain added value for real-life development, the collation of driving data should go beyond the constant average speed assumption [34]. Furthermore, driving speeds should be recorded at significantly shorter intervals using three-dimensional acceleration data. Notably, to increase EMS driving safety, there is not only a need for guidelines and supervision, but also immediate feedback. Previous studies have generated promising results, for example, from onboard safety monitoring and feedback systems, which help address potentially detrimental driving behaviors [2,4,24,30,35,36,37].

### Methodological Considerations

The strength of this study lies in its examination of real-life, whole-year data and of a large dataset of both urgent and non-urgent ambulance missions. Moreover, this study utilized an automatic ambulance tracking system based on a TETRA radio network. The system reported the speed, direction, and location at preset intervals, which were linked to digital road network data. Nevertheless, the limitation of this data is that the registered locations were often relatively inaccurate, and many locations were outside our preset limit (maximum 5 m distance from the road’s central line). These weaknesses were attributed to older TETRA terminals not utilizing modern augmented satellite navigation systems. Thus, this resulted in relatively modest accuracy levels. In addition, the accuracy may have been further reduced by data compression during transmission.

There appears to be a relatively broad variation in speeds within the speed limit. Nonetheless, this study is aimed at observing seasonal variation only, but more explanatory factors are needed to explain this variation. For further analysis, weather and visibility data should also be assessed and linked to the data. In general, the differences between summer and winter and interquartile ranges are relatively large. Accordingly, separate EMS catchment area estimates for normal speed limit periods and reduced winter speed limit periods should be considered.

The Pirkanmaa Hospital District is the second largest hospital district based on population, and eighth by area. The area is versatile, with a large central city (City of Tampere) with a high-density urban area. Moreover, it has a surrounding rural municipality with a modest population density and, especially in the northern parts of the district, relatively large low-or non-populated rural and forest regions. Thus, this area boasts a good representation of the various area types in Finland. However, the findings of this study cannot be interpreted simply by geographical features or the difference in target response times of different risk classification areas.

## 5. Conclusions

Speeding is common in both urgent A-missions and non-urgent D-missions throughout the year. The speed differences between the normal speed limit and the reduced winter speed limit periods were more evident in the urgent A-missions. In contrast, the speeds were broadly similar in non-urgent D-missions, regardless of the seasonal speed limit. More pronounced differences were observed on the smallest roads with a 30 km/h speed limit. Overall, the observation of speeding in non-urgent D-missions is a matter of patient, traffic, and work safety. Consequently, there is a need to develop stricter guidelines to increase overall EMS driving safety.

## Figures and Tables

**Table 1:** Registered driving speeds by road speed limit in the urgent ambulance A-mission group.

	Speed Limit (km/h)	*n*	Average Speed (km/h)	SD (km/h)	Lower Quartile (25%)	Median (50%)	Upper Quartile (75%)
Normal speed limit	30	1667	26.9	20.7	14.0	22.0	33.7
	40	4451	45.4	22.5	28.0	45.5	61.3
	50	7173	67.6	27.1	49.0	66.0	82.6
	60	8139	85.8	20.6	73.8	89.0	99.5
	70	5645	91.8	20.6	79.5	95.9	107.2
	80	17,343	104.2	22.8	92.3	107.2	119.9
	100	29,321	120.0	17.1	111.3	119.9	129.2
	120	1212	136.9	16.4	129.2	139.2	150.0
Winter reduced speed limit	30	1412	27.4	17.8	17.0	26.0	33.7
	40	4370	43.1	21.3	27.0	42.2	56.9
	50	6829	65.1	24.4	49.0	63.6	79.5
	60	6 88	80.9	19.7	68.5	82.6	95.9
	70	5081	86.9	19.4	76.6	89.0	99.5
	80	22,820	102.0	20.4	92.3	103.3	115.5
	100	18,689	114.3	17.3	103.3	115.5	124.4

**Table 2:** Registered driving speeds by road speed limit in non-urgent ambulance D-mission group.

	Speed Limit (km/h)	*n*	Average Speed (km/h)	SD (km/h)	Lower Quartile (25%)	Median (50%)	Upper Quartile (75%)
Normal speed limit	30	16,097	21.6	12.5	13.0	19.0	27.0
	40	66,495	30.6	14.8	19.0	31.3	40.6
	50	60,261	46.6	17.5	36.3	47.2	56.9
	60	45,181	62.0	14.9	54.8	63.6	71.1
	70	31,674	65.2	15.0	59.0	66.0	73.8
	80	64,480	78.6	16.6	71.1	82.6	89.0
	100	55,146	95.6	14.5	89.0	95.9	103.3
	120	2998	114.0	14.9	107.2	115.5	124.4
Winter reduced speed limit	30	13,304	22.5	12.6	14.0	21.0	28.0
	40	61,358	30.1	14.3	19.0	30.2	40.6
	50	52,754	45.9	16.6	36.3	47.2	54.8
	60	41,553	60.7	14.5	54.8	61.3	68.5
	70	29,124	65.3	13.3	61.3	66.0	73.8
	80	78,749	79.0	14.8	73.8	82.6	85.7
	100	33,585	93.5	14.7	85.7	95.9	103.3

**Table 3:** Distribution of registered driving speeds in non-urgent ambulance D-missions by Finnish traffic violation legislation.

Speed Limit (km/h)	% of Registered Driving Speeds		
	Within Limits (%)	Max. 20 km/h Overspeed (Administrative Traffic Violation)	>20 km/h Overspeed (Criminal Income-Based Fine)
30	90.0	7.1	3.0
40	86.9	11.3	1.8
50	79.1	14.9	6.0
60	65.8	28.5	5.8
70	86.7	11.8	1.5
80	73.9	21.9	4.2
100	77.9	20.3	1.8
120	84.9	15.0	0.1
Total	78.8	17.6	3.6

## Data Availability

Data are available upon reasonable request from the corresponding author (https://orcid.org/0000-0002-1174-8669). Map data are copyrighted to OpenStreetMap contributors and are available from https://www.openstreetmap.org. The map data contain data from the National Land Survey of Finland’s (NLS) Topographic Database and other datasets, under the NLS Open Data License.
